# Circulating Endothelial Progenitor Cells in Kidney Transplant Patients

**DOI:** 10.1371/journal.pone.0024046

**Published:** 2011-09-08

**Authors:** Giovana S. Di Marco, Peter Rustemeyer, Marcus Brand, Raphael Koch, Dominik Kentrup, Alexander Grabner, Burkhard Greve, Werner Wittkowski, Hermann Pavenstädt, Martin Hausberg, Stefan Reuter, Detlef Lang

**Affiliations:** 1 Medizinische Klinik und Poliklinik D, Universitätsklinikum Münster, Münster, Germany; 2 Institut für Anatomie, Universitätsklinikum Münster, Münster, Germany; 3 Institut für Medizinische Informatik und Biomathematik, Universitätsklinikum Münster, Münster, Germany; 4 Institut für Strahlenbiologie, Universitätsklinikum Münster, Münster, Germany; University of California Los Angeles, United States of America

## Abstract

**Background:**

Kidney transplantation (RTx) leads to amelioration of endothelial function in patients with advanced renal failure. Endothelial progenitor cells (EPCs) may play a key role in this repair process. The aim of this study was to determine the impact of RTx and immunosuppressive therapy on the number of circulating EPCs.

**Methods:**

We analyzed 52 RTx patients (58±13 years; 33 males, mean ± SD) and 16 age- and gender-matched subjects with normal kidney function (57±17; 10 males). RTx patients received a calcineurin inhibitor (CNI)-based (65%) or a CNI-free therapy (35%) and steroids. EPC number was determined by double positive staining for CD133/VEGFR2 and CD34/VEGFR2 by flow cytometry. Stromal cell-derived factor 1 alpha (SDF-1) levels were assessed by ELISA. Experimentally, to dissociate the impact of RTx from the impact of immunosuppressants, we used the 5/6 nephrectomy model. The animals were treated with a CNI-based or a CNI-free therapy, and EPCs (Sca+cKit+) and CD26+ cells were determined by flow cytometry.

**Results:**

Compared to controls, circulating number of CD34+/VEGFR2+ and CD133+/VEGFR2+ EPCs increased in RTx patients. There were no correlations between EPC levels and statin, erythropoietin or use of renin angiotensin system blockers in our study. Indeed, multivariate analysis showed that SDF-1 – a cytokine responsible for EPC mobilization – is independently associated with the EPC number. 5/6 rats presented decreased EPC counts in comparison to control animals. Immunosuppressive therapy was able to restore normal EPC values in 5/6 rats. These effects on EPC number were associated with reduced number of CD26+ cells, which might be related to consequent accumulation of SDF-1.

**Conclusions:**

We conclude that kidney transplantation and its associated use of immunosuppressive drugs increases the number of circulating EPCs via the manipulation of the CD26/SDF-1 axis. Increased EPC count may be associated to endothelial repair and function in these patients.

## Introduction

Endothelial dysfunction is a typical finding in chronic kidney disease (CKD). It contributes to accelerated arteriosclerosis and impaired angiogenesis and, therefore, to high cardiovascular morbidity and mortality in these patients. However, after renal transplantation (RTx) endothelial function improves, even though substantial dysfunction is still observed in these patients [1]–[3]. Thus, it is not surprising that endothelial damage, as a process of the whole vasculature, is an important feature of chronic allograft nephropathy [3].

Interestingly, these vascular lesions can be repaired by i) migration and proliferation of endothelial cells contiguous to the lesions or by ii) the so-called endothelial progenitor cells (EPCs) [4]. These cells reside in the bone marrow and are mobilized to the peripheral blood upon stimulation. Stimuli include tissue ischemia and local release of cytokines and growth factors [5]. The stromal cell-derived factor 1 alpha (SDF-1) is one of these chemokines that serve as chemoattractant for stem/progenitor cell populations [6].

Patients with advanced renal failure were shown to have not only significant lower EPC numbers compared to healthy controls but, in addition, impaired EPC function [7]. EPC number and function can be restored by initiation of dialysis or kidney transplantation, procedures at least partially restoring or imitating renal function [8]–[10].

During the transformation process of EPCs into mature endothelial cells, human EPCs express different surface markers at distinct stages including CD133, CD34 and vascular endothelial growth factor receptor 2 (VEGF R2) [11]. Circulating EPCs seem to prefer to locate at the sites of vascular lesions, thereby, contributing essentially to both reendothelialization and revascularization [12]. Thus, EPCs are critically involved in maintaining the integrity of the endothelium and repairing vascular damage [13].

Immunosuppressive treatments of patients after RTx may directly affect the endothelial function [14], [15]. However, the exact role of EPC and the EPC count in recipients of renal allografts is still controversial. Therefore, the aim of the present study was to determine i) the number of EPCs in stable renal allograft recipients and ii) the EPC count association with different immunosuppressive agents especially the comparison of calcineurin inhibitor (CNI)-based and CNI-free therapies. Moreover, we provided a current literature review on studies regarding EPC in RTx.

## Results

### Human study

Clinical data of the study subjects are summarized in Table S1. All patients received medication, including immunosuppressive drugs, statins, antihypertensive drugs, and/or erythropoietin. We studied a total of 52 stable kidney transplant patients and 16 gender- and age-matched subjects. 68% (38/56) of the patient cases were on CNI (cyclosporine, 90.6±3.2 ng/ml, or FK506, 8.7±3.1 ug/ml), and 32% (18/56) were mostly on mycophenolate mofetil (MMF, 3.6±1.7 ug/ml) and sirolimus (CNI-free therapy). At the time of blood collection, most of the patients given a CNI-based immunosuppression used a FK506 regimen (19/38) followed by FK506+MMF (11/38); most recipients treated with a CNI-free regimen received MMF and steroids (16/18). The glomerular filtration rate (eGFR) estimated by the MDRD (Modification of Diet in Renal Disease) formula was in all graft recipients above 40 ml/min/1.73 m^2^ and in controls above 60 ml/min/1.73 m^2^, respectively. The average time period between RTx and blood collection was 59±53 months (Mean ± SD). A possible interrelation between waiting time since surgery and EPC count was ruled out by univariate regression analysis (data not shown).

**Figure 1:**
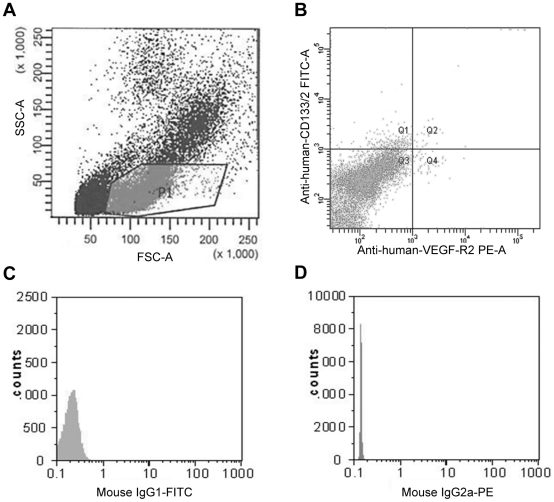
Representative flow cytometry analysis of an EDTA-blood sample from a patient. Circulating EPCs were identified by the expression of cell surface antigens, such as CD34^+^, CD133^+^, and VEGF-R2^+^. A) Density plot with forward (FSC) and side light scatter (SSC). P1-gate was selected for further analysis. B) Density plot of PE-conjugated anti-VEGF-R2 antibody versus FITC-conjugated anti-CD133 antibody. Cells double positively stained for VEGF-R2 and CD133 (quadrant Q2) represent CD133^+^ endothelial progenitor cells (CD133^+^/VEGFR2^+^ EPCs). C) Mouse-IgG1-FITC negative control and D) Mouse-IgG2a-PE negative control.

**Figure 2:**
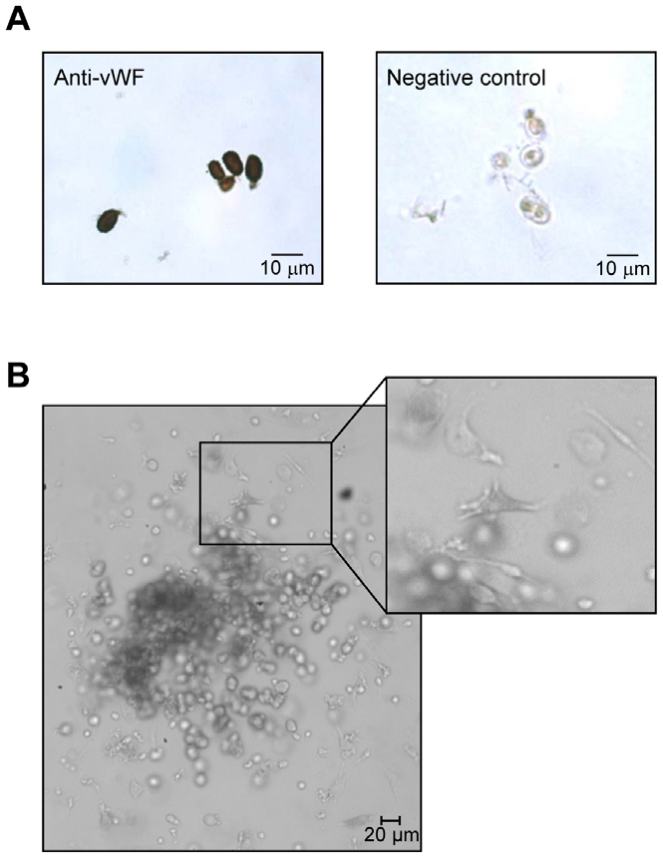
Isolation and characterization of CD133+/VEGFR2+ cells. Cells sorted by FACS were further characterized by the expression of a specific endothelial cell marker or cultured in a human methylcellulose base media (A and B, respectively). A) CD133^+^/VEGFR2^+^ cells were immunohistochemically stained with an antibody against von Willebrand Factor (vWF). Negative control: omission of the primary antibody. B) Phenotypically, colonies formed by these cells in methylcellulose base media show the typical shape of early EPC-colonies with round immature cells in the center and dendritic or spindle cell-shaped peripheral cells (see magnification).

Blood samples were obtained as part of a routine diagnostic or screening procedure. They were analyzed within 1 hour. Figure 1 shows a representative density plot of the flow cytometric analysis of a patient's sample. CD133+/VEGFR+ cells were further characterized immunohistochemically by the expression of von Willebrand Factor (vWF) and their phenotypic definition as endothelial precursors was confirmed by EPC outgrow in culture (Figure 2A and B).

**Figure 3:**
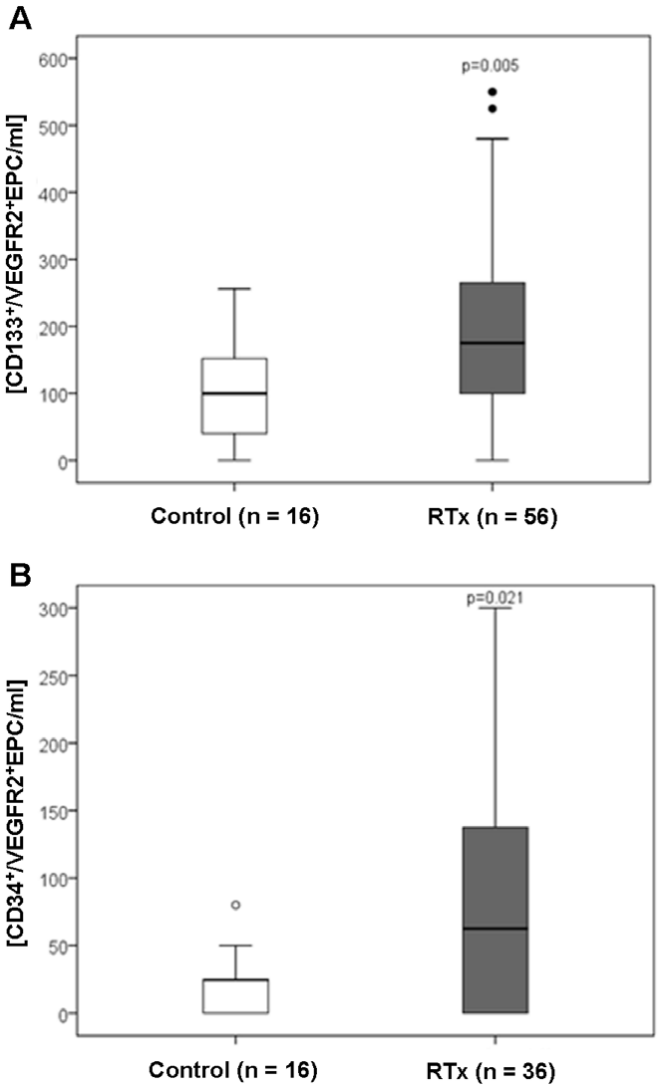
Circulating levels of endothelial progenitor cells (EPC) in renal transplant recipients. EPC levels were directly quantified from whole blood taken from control subjects and patients (RTx) by flow cytometry, which identifies EPCs according to the expression of cell surface antigens, such as (A) CD133^+^ and VEGF-R2^+^; and (B) CD34^+^ and VEGF-R2^+^. P value compared to control group is indicated (Mann-Whitney test).

**Figure 4:**
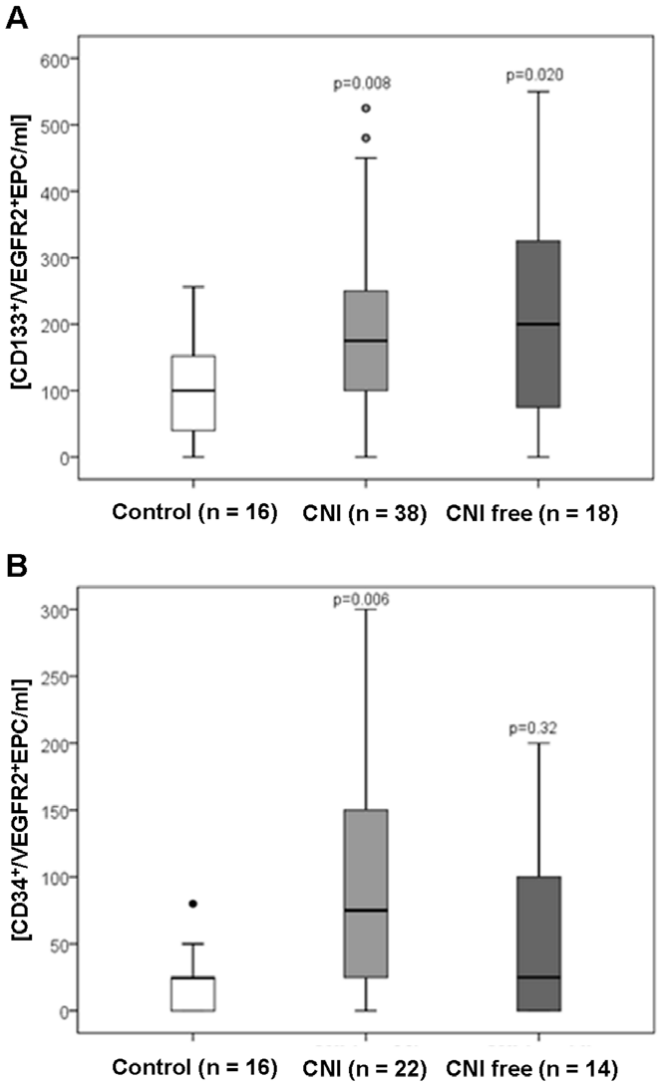
Circulating levels of EPC in renal transplant recipients according to their immunosuppressive therapy. (A) CD133^+^ and VEGF-R2^+^; and (B) CD34^+^ and VEGF-R2^+^. CNI, calcineurin inhibitor. P value compared to control group is indicated (Mann-Whitney test).

Circulating EPC – both, CD133^+^/VEGFR2^+^ and CD34^+^/VEGFR2^+^ EPCs – number is increased in RTx recipients when compared to controls (Figure 3). To elucidate the effect of immunosuppressive therapy on EPC count, CNI-based and CNI-free regimens were compared (Figure 4). Compared to controls, the number of circulating CD133^+^/VEGFR2^+^ cells increased in RTx patients independently of the immunosuppressive regimen used (Table S1 and Figure 4), while CD34^+^/VEGFR2^+^ EPCs increased only in CNI-treated patients only.

**Figure 5:**
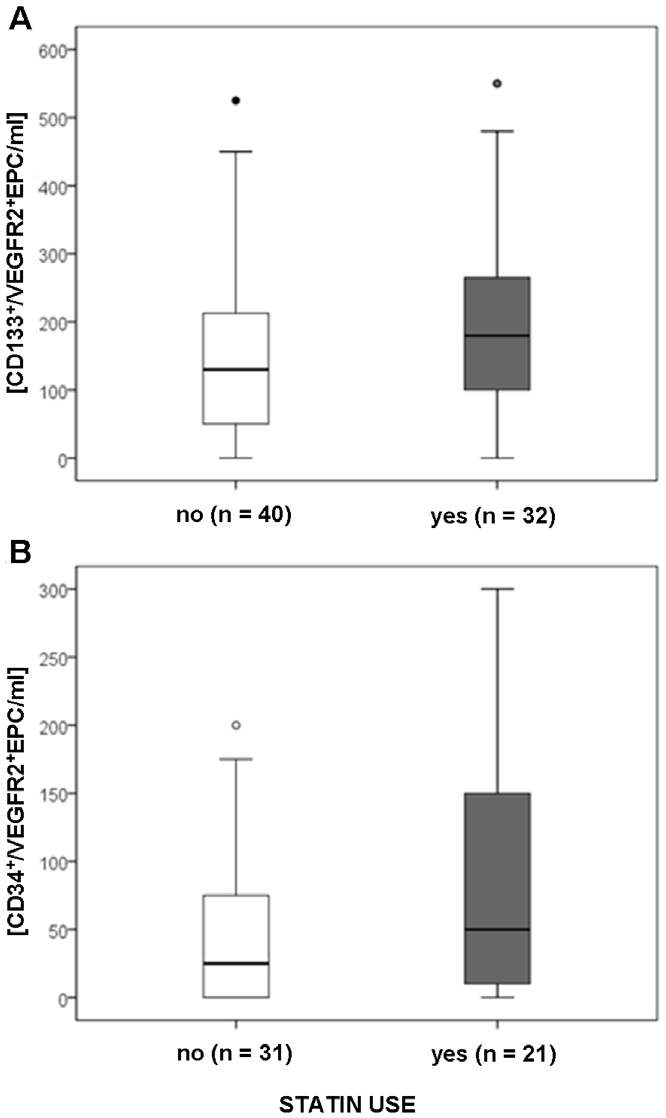
Circulating levels of EPC in renal transplant recipients are not associated with statin use. A) CD133^+^/VEGFR2^+^ EPCs; B) CD34^+^/VEGFR2^+^ EPCs.

Besides the immunosuppressive therapy, we analyzed if renal function (eGFR), diabetes mellitus and statin use interfere with the EPC count. In multivariate analysis we could not show any relation between eGFR or diabetes mellitus with circulating EPC number (Table 1). RTx recipients receiving statins presented 50.0 (5.0–150.0; n = 21) CD34^+^/VEGFR2^+^EPCs/ml and 179.5 (100.0–272.5; n = 32) CD133^+^/VEGFR2+EPC/ml (results are median and interquartile range), respectively; while RTx recipients without statin therapy tended to lower EPC counts/ml: 25.0 (0.0–75.0; n = 31) CD34^+^/VEGFR2^+^EPCs/ml and 130.0 (50.0–218.75; n = 40) CD133^+^/VEGFR2+EPC/ml, respectively (Figure 5). However, these differences did not reach statistical significance.

**Table 1:** Relation of different parameters with circulating EPC numbers.

	CD133^+^EPC	
	No stand. B	p-value
**Full Model (backward selection):**		
RTx (yes or no)	0.157	0.760
eGFR (ml/min/1.73 m^3^)	−0.001	0.789
CNI (yes or no)	0 037	0.908
CNI free (yes or no)	−0.037	0.898
Statin (yes or no)	0.265	0.239
Antihypertensive therapy (yes or no)	0.135	0.505
Diabetes mellitus (yes or no)	−0.119	0.644
SDF-1 alpha (pg/ml)	0.001	0.020
**Result:**		
**SDF-1**	0.001	0.000

Multivariate analysis. CD133^+^EPC was transformed to natural logarithm. B, no standardized regression coefficient beta. CNI, calcineurin inhibitor; eGFR, estimated glomerular filtration rate; SDF-1, stromal cell-derived factor 1 alpha; RTx, Kidney transplantation.

To investigate putative mechanisms in EPC mobilization, we measured plasma levels of SDF-1 (Table S1). In RTx, elevated EPC number was accompanied by increased SDF-1 levels. Notably, multivariate regression analysis confirmed that plasma SDF-1 levels were independently associated with circulating EPC number (Table 1).

### Animal study

**Figure 6:**
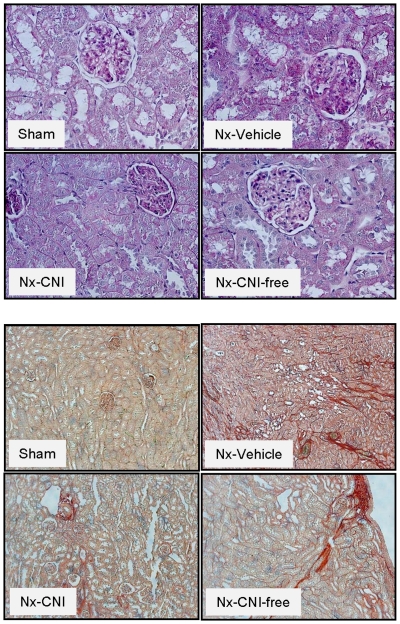
Representative renal histologies. Histological changes were examined by light microscopy in paraffin-embedded tissue with periodic acid-Schiff (PAS) (upper panels; magnification 40×) and picro Sirius red (lower panels; magnification 10×) stainings. Sham: sham animals; 5/6 Nx: nephrectomy; CNI: calcineurin inhibitor (cyclosporine A 5 mg/kg/day); CNI free: mycophenolate mofetil 30 mg/kg/day.

To distinguish the impact of RTx from the impact of immunosuppressive drugs on the number of circulating EPCs - as well as to avoid potential confounders, such as concomitant diseases and medications present in human patients - we decided to use an additional experimental model. Since our RTx patients presented a 59 to 62% reduction in the GFR in comparison to controls (Table S1), we have chosen the 5/6 nephrectomy (Nx) model that presents a similar impairment of the renal function (50%-reduction of the creatinine clearance). Based on the rat functional data assessed 14 days after surgery (Table 2) and histological analysis (Figure 6), we can state that 5/6 Nx leads to decreased renal function (increased serum creatinine and blood urea nitrogen and decreased creatinine clearance) and histological changes in the kidneys such as interstitial fibrosis, glomerular sclerosis, and tubular atrophy. However, treatment with cyclosporine A and MMF do not further deteriorate renal function or kidney injury, but significantly ameliorated albuminuria/proteinuria.

**Table 2:** Animal functional data (day 14 after surgery).

	Sham	5/6 Nx		
		Vehicle	CNI	CNI free
	n = 8	n = 6	n = 5	n = 5
**Body weight**	310±15	302±18	309±10	270±5
**Urine volume (ml/24 h)**	17±2	29±4	28±8	32±5
**Serum creatinine (mg/dl)**	0.2±0.09	0.5±0.06*	0.5±0.06*	0.4±0.01
**Blood urea nitrogen (mg/dl)**	18±2	36±6*	50±8*	34±3*
**Urinary Albumin (mg/mg Cr)**	0.10±0.03	4.1±1.13*	0.8±0.58^#^	0.4±0.08^#^
**Urinary Protein (mg/mg Cr)**	0.45±0.06	8.8±2.7*	2.2±0.43^#^	1.25±0.10^#^
**CrCl (ml/min/100 g)**	0.9±0.04	0.4±0.07*	0.5±0.07*	0.5±0.07*
**BUN-Cl (ml/min/100 g)**	0.4±0.05	0.2±0.04*	0.2±0.03*	0.3±0.02
**(CrCl+BUN)/2-Cl (ml/min/100 g)**	0.6±0.05	0.3±0.05*	0.3±0.05*	0.4±0.02*

Results are mean ± SEM. BUN, blood urea nitrogen; Cr, creatinine, CrCl, creatinine clearance. Sham: sham animals. 5/6-nephrectomised rats (Nx) were treated (i.p) with: saline (vehicle); cyclosporine A 5 mg/kg/day (calcineurin inhibitior; CNI) and mycofenolat mofetil 30 mg/kg/day (CNI free). Results are mean ± SEM.

*P<0.05 compared to Sham.

**Figure 7:**
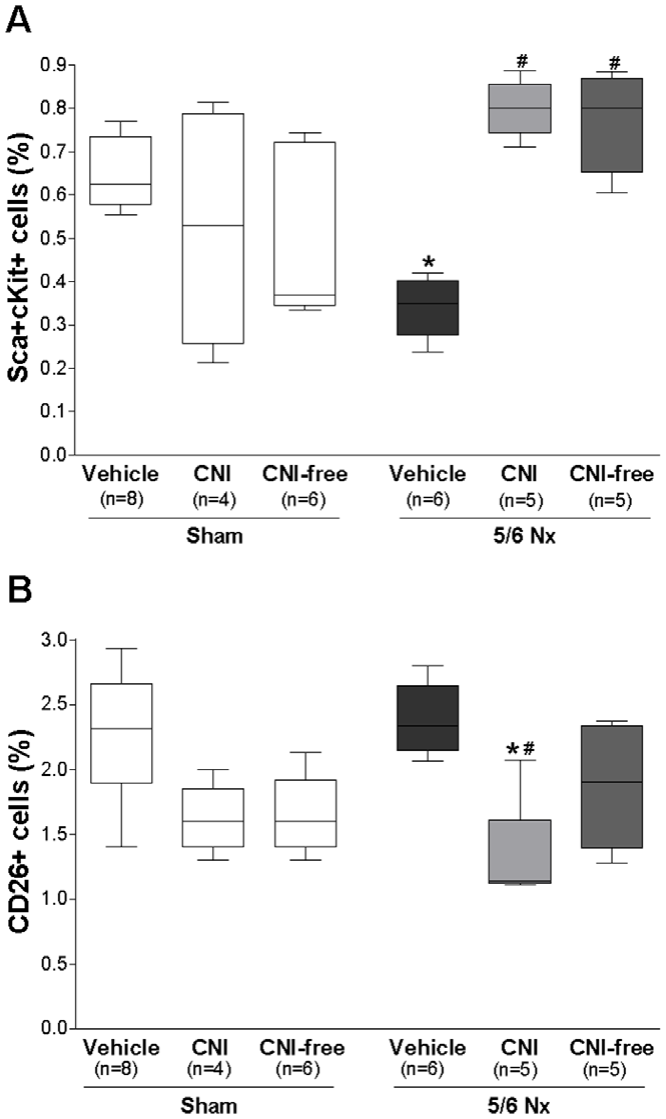
Effect of decreased renal function and immunosuppressive agents on circulating levels of progenitor cells in rats. Progenitor cells were defined by the surface expression of stem cell antigen-1 (Sca-1) and c-Kit antigens. The number of circulating progenitor cells (A) and CD26^+^ cells (B) was determined by flow cytometry 14 days after surgery/treatment. Sham: sham animals; 5/6 Nx: nephrectomy; CNI: calcineurin inhibitor (cyclosporine A 5 mg/kg/day); CNI free: mycophenolate mofetil 30 mg/kg/day. Results are mean ± SEM. ^*^P<0.05 compared to Sham; ^#^P<0.05 compared to vehicle.

Progenitor cells were defined by the surface expression of stem cell antigen-1 (Sca-1) and c-Kit antigens. This cell population represents highly immature cells that account for a small fraction of circulating mononuclear cells and include endothelial-committed precursors involved in compensatory angiogenesis at ischemic sites [16]. As expected, 5/6 Nx rats presented decreased number of circulating Sca^+^cKit^+^ cells when compared to sham-operated rats. CNI- and CNI-free-treated rats presented not only an increased number of progenitor cells in comparison to vehicle-treated 5/6 Nx rats (Figure 7A), but also in comparison to sham-operated rats (0.80%±0.04 vs. 0.61%±0.05, mean ± SEM, sham vs. CNI, Mann Whitney test P = 0.02; 0.77%±0.05 vs. 0.61%±0.05, sham vs. CNI-free, P = 0.08). CNI or CNI-free therapy given to sham rats did not interfere with EPC numbers.

Attenuation of the CD26 system can lead to increased concentration of SDF-1. Rats with renal failure and CNI treatment had lower circulating CD26^+^ cells number than sham and vehicle-treated 5/6 Nx rats. In CNI-free-treated rats the CD26^+^ cells number was slightly lower (Figure 7B). These results are in agreement with previous results of our group that show increased SDF-1 levels in CNI-treated rats [17].

## Discussion

Few studies have yet reported on EPC counts in RTx (Table S2). Previous studies demonstrated reduced EPC levels in CKD [7], whereas graft function seems to influence EPC number and function in RTx recipients [10], [18]–[20]. We herein show that RTx recipients on immunosuppressive medication present increased number of circulating EPCs when compared to controls subjects. Furthermore, EPC levels were found to be independently associated with plasma SDF-1 levels, a chemokine responsible for the homing and mobilization of progenitor cells.

EPC can be characterized by hematopoietic stem cell markers (clusters of differentiation) such as CD34 or CD133 combined with the expression analysis of an endothelial surface marker (VEGFR2 or KDR, von Willebrand factor, VE cadherin, CD146, CD31), uptake of Dil-acetylated lipoprotein, and lectin binding [5], [21]. CD34 is an early marker expressed by bone marrow cells and EPCs, and also by endothelial and hematopoietic cells. Co-expression of CD34 and VEGFR2 has been used in various studies to identify circulating progenitor cells [22], [23]. Alternatively and more recently, CD133, a marker of more immature hematopoietic stem cells, was used for identification of these cells. Double staining for CD133 and VEGFR2 performs better than CD34 staining only to identify immature progenitor cells because CD34^+^/VEGFR2^+^ cells may also act as reserve cells embedded in the vessel wall or as endothelial cells with a mature phenotype. Finally, it is commonly agreed that the CD133^+^/VEGFR2^+^ cell fraction is a population with characteristics of EPCs [24], [25]. Thus, we analyzed 3 cellular markers of EPCs: CD34, CD133, and VEGFR2 in the present study.

To exclude influences of blood pressure, anti-hypertensive medication and other co-morbidities, we have chosen a control group of mostly hypertensive patients with normal kidney function for comparison to RTx recipients. Even though a higher mean blood pressure was associated with lower EPC counts in a study with RTx recipients [20], hypertension itself seems not to influence the number or functional activity of EPC [26]–[28], and all patients recruited in our study achieved normotension by treatment.

It has been shown that the graft function is an important determinant of EPC number and function in RTx recipients [10], [18]. De Groot et al. found a positive correlation between renal function and EPC level [10]. Interestingly, the simple removal of the uremic state contributed substantially to the restoration of EPC count in these patients [10]. Taken all currently available studies on EPC and RTx together [10], [18]–[20], [29], one study found a decreased EPC count, and four studies demonstrated that RTx recipients present EPC levels comparable to healthy subjects (Table S2). Two studies present additional functional data. While Soler et al. [19] assessed reduced EPC function, Herbig et al. [18] observed improved EPC function after RTx when compared to controls. De Groot et al. have shown that the number of mononuclear cell-derived EPCs in culture is similar in RTx and healthy controls. Nevertheless, the level of CD34+ hematopoietic progenitor cells are significantly higher in patients than in controls [10]. To note, CD34 positivity alone or in different combinations has also been used to define circulating EPCs [30]. The results obtained with the latter method are in full agreement with our findings, since we have observed increased CD34+/VEGFR2+ and CD133+/VEGFR2+ cell levels in RTx patients. These contradictory results might be related to the different methodologies and markers employed in the study of EPC number. Moreover, different studies might include patients with different cumulative cardiovascular risk profiles. This might be relevant, given that the EPC number can be affected negatively or positively by individual cardiovascular risks [30].

Three factors could have influenced the EPC number in RTx recipients: the immunosuppressive therapy, RAS blockade and statin use. Indeed, we did not find a correlation between EPC levels and statin use or RAS blockade, drugs which have previously been reported to increase EPC numbers [20], [31], [32]. RAS blockade did not differ between groups and, even though RTx patients who received statins presented an increased EPC number compared to controls, so did all the other patients in our study. In agreement with our findings, previous results have also described the inability of statins to influence EPC count in end-stage renal patients [28], [33]. Finally, in our study population, RTx presented increased CD133^+^/VEGFR2^+^EPC number independently of the immunosuppressive drugs, while CD34^+^/VEGFR2^+^EPC cells were improved only in CNI-treated patients.

To dissociate the impact of kidney transplantation itself and concomitant medication from the impact of immunosuppressive drugs (CNI vs. CNI-free) on the number of circulating EPCs, we have employed a defined animal model, the 5/6 nephrectomy to minimize confounders. 5/6 nephrectomy led to a decreased renal function (around 50% reduction in the GFR) which was comparable to the decreased renal function of RTx patients. 5/6 rats received no other drugs besides cyclosporine A or mycophenolate mofetil. Interestingly, as seen in uremic patients, vehicle-treated 5/6 Nx rats presented decreased circulating EPC counts, confirming that decreased renal function is directly associated to decreased EPC numbers. Even under this detrimental condition (uremia), CNI and CNI-free therapies improved the number of circulating progenitor cells in comparison to vehicle-treated 5/6 rats.

However, both immunosuppressants were not able to increase EPC number in sham-operated rats. These results suggest that, besides the immunosuppressive therapy, ischemic stress is also necessary to affect progenitor cell count in our model [34]. In agreement with our results, Wang et al. have already shown that without ischemia, treatment with cyclosporine A does not lead to significant differences in the circulating levels of progenitor cells, as well as in the concentrations of EPC-associated cytokines [6]. In addition, by using the same model, we have recently shown that CNI-treated rats present not only increased mobilization of stem/progenitor cells, but also that these cells are able to incorporate into sites of injury, thereby conferring cardioprotection in these rats [17].

EPCs participate in the repair of endothelial dysfunction [8], a process divided into 3 different stages: mobilization from bone marrow, homing into the sites of injury, and incorporation into the endothelium [5]. Cytokines released by e.g. damaged tissues mobilize EPCs, which in turn migrate and promote local neovascularization. Recent studies indicate that the interplay between SDF-1 and EPC is the main driving force behind the mobilization and recruitment process [35].

SDF-1 is constitutively expressed by most organs in the body. Interestingly, after kidney injury, its level is not only increased in the kidney, but also in the circulation [36]. Herein, we have shown that plasma SDF-1 levels are increased in RTx patients. Elevated circulating SDF-1 concentrations can result from inhibition of the CD26 (dipeptidylpeptidase IV), a membrane-bound extracellular peptidase with the ability to cleave the cytokine [6]. In the circulation, lymphocytes are the main source for CD26 [37]. We and others have already demonstrated that kidney recipients receiving immunosuppressant drugs exhibit lower CD26 activity/availability when compared to healthy individuals (Figure S1, Table S3, Ref. [38]), and that CsA treatment decreases the number of circulating CD26^+^ cells in the peripheral blood of rats [6], [17]. In addition, in the hindlimb ischemia model, the same effects (reduced circulating CD26+ cells/decreased enzymatic activity) were associated to cyclosporine A therapy [6]. Altogether, these results suggest that the use of an immunosuppressive therapy lowers the CD26/dipeptidyl peptidase IV enzymatic activity in peripheral blood, therefore avoiding SDF-1 inactivation and promoting its increase in the circulation. Increased serum SDF-1 concentration increases EPC mobilization from the bone marrow to the circulation. EPCs are then able to home into sites of tissue hypoxia and/or damage.

Finally, a strong evidence for the functional relevance of EPC for the positive effects of CNI/CNI-free on endothelial repair in the 5/6 model is the fact that treated rats presented reduced urinary albumin-to-creatinine and protein-to-creatinine ratios in comparison to vehicle-treated animals. It is well established that albuminuria/proteinuria reflects not only glomerular, but also generalized endothelial dysfunction, which explains its prognostic value (a sensitive marker) for renal and cardiovascular risks [39], [40].

In conclusion, we found that kidney transplantation and its associated use of immunosuppressive drugs lead to improved number of circulating EPCs. The nature and size of our study do not permit us to determine whether high levels of these cells can affect endothelial function in RTx cases. Rather, we can speculate that this increase in EPC count is associated with increased SDF-1 levels, suggesting increased endothelial repair and function in these patients.

## Materials and Methods

### Characteristics of patients and control subjects

Fifty two kidney transplant patients were included from the Transplantation Unit of the Department of Internal Medicine D, University Clinics Münster, Germany. As the most of the patients were hypertensive (70%), 16 age-matched subjects - of whom 11 with essential hypertension and normal kidney function - served as a control group to exclude implications of blood pressure as well as of antihypertensive treatment. Hypertension was controlled by medication in both groups.

EDTA-blood was obtained from all control subjects and patients. The blood samples of the patient cases were collected 50±46 and 77±62 months (mean ± SD) after kidney transplantation in both, CNI- and CNI free-groups, respectively.

The protocol was approved by the medical ethical committee of the University Clinics Münster (permit number 4IX Kosch-Lang). Written informed consent was obtained from all patients and control subjects.

### Flow cytometry of human circulating endothelial progenitor cells (EPC)

The total number of circulating EPCs was analyzed by flow cytometry as previously described [25], [41]. EDTA-blood samples taken from controls and patients (four aliquots of 100 µl) were incubated for 30 minutes in the dark with the following antibody combinations: 1) PE-conjugated mouse IgG2a (Serotec, Germany)+FITC-conjugated mouse IgG1 (Serotec, Germany)+Streptavidin-PECy5; 2) PE-conjugated anti-human VEGF-R2 (R&D Systems, Germany)+CD133-FITC+7-AAD; 3) PE-conjugated anti-human VEGF-R2+CD133-PECy5+FITC-conjugated anti-human CD45 (R&D Systems, Germany); and 4) PE-conjugated anti-human VEGF-R2+FITC-conjugated anti-human CD34 (Serotec, Germany)+7-AAD. To get this combination, Biotin-conjugated anti-human CD 133 (Miltenyi Biotec, Germany) was used followed by an FITC-conjugated anti-Biotin (Miltenyi Biotec, Germany) or a PECy5-conjugated Spreptavidin (eBioscience, United Kingdom) secondary antibody, respectively, as well as the viability staining reagent 7-amino actinomycin D (7-AAD; eBioscience, United Kingdom). Isotype-matched antibodies served as negative controls. CD45 was used as an internal control for an equal number of white blood cells in each sample, while 7-AAD staining was used to eliminate dead cells by flow cytometry.

After staining, cells were washed with PBS, lyzed with IO-Test 3 lyzing solution according to the manufacturer's instructions (Beckmann Coulter), and resuspended in PBS (1 ml). The double-labeled samples were then analyzed on a flow cytometer equipped with an electronic volumeter, which allows an exact measurement of the volume of the specimen aspired by the flow cytometer. A fixed volume of 200 µl was used, which, by the given dilution factor, allows the analysis of about 100.000 white blood cells in each single measurement. Thus the leukocyte, respectively the EPC, concentration was provided directly by the instrument (PAS III flow cytometer, equipped with a 20-mW 488-nm argon ion laser and 5 PMT's: FSC, SSC, FL1-3; Partec GmbH, Germany) [42].

The threshold was set at the lower end of the forward scatter. Gates were set at forward scatter (FSC) and sideward scatter (SSC), including mononuclear cells and excluding PMNLs. Cells inside this gate were further analyzed with regard to their fluorescence properties. A gate was set around the region containing the double positively stained cells for the combinations: CD34-FITC/VEGF-R2-PE; CD34-FITC/CD133-PE; and CD34-FITC/CD45-PE. EPC number was determined by means of double positively stained mononuclear cells for VEGF-R2 and CD133 or VEGF-R2 and CD34 (CD133^+^/VEGFR2^+^ and CD34^+^/VEGFR2^+^ cells, respectively) [43].

The reproducibility and variability of the method per patient over time had been previously determined by Rustemeyer et al. Moreover, our method presented high correlation with a cell culture method where the cytometrically purified stem cells (EPC) demonstrated their colony forming capacity [25]. The CD133+ and VEGF-R2+ cells from the cell sorter (FACSAria, BD Biosciences, USA) were cultured in a human methylcellulose base media (R&D Systems, USA) supplemented with β-EGF, IL-3 and SCF. All cell cultures were maintained at 37°C with 5% CO2 in a humidified atmosphere. After 2 weeks colonies were counted by two or three independent investigators. These colonies showed the typical shape of early EPC-colonies with round immature cells in the center and dendritic or spindle cell-shaped peripheral cells.

For further characterization cytospins of colonies were made. Cells were stained with 4′6-diamidino-2-phenylindole (DAPI, Sigma-Aldrich, Germany) and unconjugated monoclonal antibodies against von Willebrand Factor (vWF; Dako, Denmark). Immunodetection was visualized by FITC-labeled goat-anti-mouse-antibody (Dako, Denmark).

In addition, sorted CD133^+^/VEGFR2^+^ cells were directly transferred to a glass slide coated with poly-L-lysine (Sigma Aldrich, Germany), fixed with 4% paraformaldehyde and subsequently submitted to immunohistochemical analysis by using a polyclonal antibody against vWF (dilution 1∶100; Abbiotec, USA) and HRP-conjugated secondary antibody (dilution 1∶200; Vector laboratories, USA). Omission of the primary antibody was used as negative control.

### CD26 and SDF-1 determination

CD26 and stromal-derived factor 1 alpha (SDF-1) levels were measured in patients' and controls' plasma by using commercial ELISA kits (human DPPIV/CD26 and human CXCL12/SDF-1 alpha immunoassay, respectively, R&D Systems). Samples for CD26 determination were 100-fold diluted in Calibrator Diluent according to manufacturer's specifications, while SDF-1 determination does not require dilution. In both assays, the antibodies were raised against the human recombinant factors.

### Animal model of renal disease: 5/6 nephrectomized rat

Renal disease was induced in Sprague Dawley rats by 5/6-resection of renal tissue as previously described [44], [45]. Experiments were approved by a governmental committee on animal welfare (Landesamt für Natur, Umwelt und Verbraucherschutz Nordrhein-Westfalen, permit number 8.87-50.10.36.08.230) and were performed in accordance with national animal protection guidelines. In short, the 5/6-nephrectomy involved midline incision to remove the right kidney and ligation of branches of the left renal artery to infarct approximately 2/3 of the kidney mass. Surgery was performed under general anesthesia (ketamine (100 mg/kg)/xylazine (10 mg/kg)). Further ketamine was supplemented as necessary. Sham operation consisted of decapsulation of the right kidney. After surgery, the rats were randomized into 4 groups: 1) Sham+vehicle (Sham, n = 8); 2) 5/6-nephrectomized rats+vehicle (5/6 Nx, n = 6); 3) 5/6 Nx+cyclosporine A 5 mg/kg/day (CNI, n = 6); and 4) 5/6 Nx+mycophenolate mofetil 30 mg/kg/day (CNI free, n = 5). The treatment started on the day of surgery and lasted for 14 days. All drugs and saline were applied intraperitoneally (i.p.). At day 13, rats were housed in metabolic cages for 24 hours. Blood (EDTA-blood and serum) was collected by puncturing the tail vein. Whole EDTA-blood was immediately used for flow cytometry analysis. Urine and serum samples were subsequently analyzed for protein (Bradford Blue; BioRad Laboratories, Germany), creatinine (enzymatic assay; Creatinine-Pap, Roche Diagnostics, Germany), blood urea nitrogen (BUN, urease-GLDH method), and electrolytes (ISE) on a Roche Diagnostic analyzer (Modular P, Roche Diagnostics). Albuminuria was detected by using the Nephrat ELISA Kit (rat urinary albumin, Exocell). At the end of the experiment, rats were sacrificed by decapitation under anesthesia with isoflurane (2-chloro-2-(difluoromethoxy)-1,1,1-trifluoro-ethane). The kidney was excised, fixed in 4% buffered formaldehyde and embedded in paraffin. Five-µm thick sections were then cut, deparaffinised, rehydrated with graded ethanol, and stained with periodic acid Schiff (PAS) and picro-sirius red (fibrosis staining).

### Flow cytometry of rat circulating stem/progenitor cells

Circulating stem/progenitor cells and CD26^+^ cells were analyzed by flow cytometry as previously described [17]. Briefly, 100 µl of EDTA-blood samples obtained from the tail vein were incubated at 4°C for 30 minutes in the dark with the following antibody combinations: 1) IgG2b-PE+IgG1-FITC; 2) PE-conjugated anti-mouse Sca-1 (Cederlane, Canada)+FITC-conjugated anti-mouse c-Kit (BD Pharmingen); 3) PE-conjugated anti-mouse Sca-1 (Cederlane, Canada)+biotin-conjugated anti-rat CD31 (BD Pharmingen)+Streptavidin-FITC (BD Pharmingen); and 4) PE-conjugated anti-rat CD26 antibody (BD Pharmingen). Isotype-matched antibodies served as negative controls. After staining, cells were washed with PBS, lyzed with IO-Test 3 lyzing solution according to the manufacturer's instructions (Beckmann Coulter), and resuspended in PBS (∼1 ml). Samples were analyzed on a BD FACSCanto II (BD Biosciences). Gates were set at forward scatter (FSC) and sideward scatter (SSC), including lymphocytes and excluding monocytes and granulocytes. Cells inside this gate were further analyzed with regard to their fluorescence properties. Data were processed using the BDFACSDiva 6.0 Software (BD Biosciences) and analyzed using FlowJo (TreeStar).

### Statistical analysis

Analyses were performed with the PASW, Version 18.0 (SPSS Inc., Chicago, IL). Non-normal data are presented as median and interquartil range; data found to be normally distributed are presented as means ± SD. The Mann-Whitney test and Kruskal-Wallis test were used to compare two or all three groups, respectively. Variables based on proportions were analyzed by chi-square test. Multivariate regression analyses were performed to assess associations between CD133^+^EPC number and other parameters with regards to potentially confounding factors. Results are described as regression coefficient Beta (Stand. B). The two-sided p<0.05 was considered to reflect statistical significance.

Experimental data is presented as mean ± SEM. Comparison among groups was performed by Kruskal-Wallis test. A level of P<0.05 was accepted as statistically significant. Analyses were performed using GraphPad Prism version 4.0.

## Supporting Information

Figure S1 Concentration of plasma CD26 (A) and stromal cell-derived factor 1 alpha (SDF-1) (B) in control and renal transplant patients according to their immunosuppressive therapy regimen. CNI: calcineurin inhibitor. The clinical characteristics of this specific control and patient population are given in Table S3. Results are mean ± SEM. P value compared to control group is indicated (Krulkal-Wallis test).
(PDF)

Table S1 Clinical characteristics of kidney transplant patients.
(PDF)

Table S2 Available studies on endothelial progenitor cells (EPCs) after kidney transplantation (RTx).
(PDF)

Table S3 Clinical characteristics of control and Kidney transplant patients related in Figure S1.
(PDF)
