# Supplementary figures and images for: Circulating Endothelial Progenitor Cells in Kidney Transplant Patients
Source: PLoS One. 2011 Sep 8;6(9):e24046. doi: 10.1371/journal.pone.0024046 (PMC3169568; doi:10.1371/journal.pone.0024046)

Figure S1

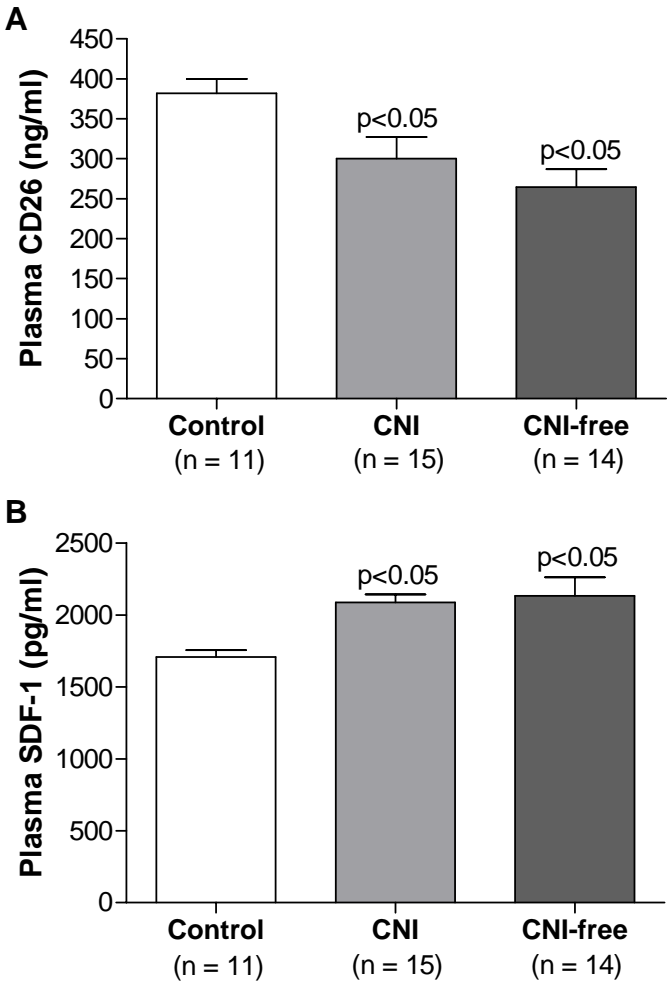

Supplement: Figure S1 — Concentration of plasma CD26 (A) and stromal cell-derived factor 1 alpha (SDF-1) (B) in control and renal transplant patients according to their immunosuppressive therapy regimen. CNI: calcineurin inhibitor. The clinical characteristics of this specific control and patient population are given in Table S3. Results are mean ± SEM. P value compared to control group is indicated (Krulkal-Wallis test). (PDF) [file pone.0024046.s001.pdf]
