# Supplementary material for: Circulating Endothelial Progenitor Cells in Kidney Transplant Patients
Source: PLoS One. 2011 Sep 8;6(9):e24046. doi: 10.1371/journal.pone.0024046 (PMC3169568; doi:10.1371/journal.pone.0024046)
Supplement: Table S1 — Clinical characteristics of kidney transplant patients. (PDF) [file pone.0024046.s002.pdf]

Table S1. Clinical characteristics of kidney transplant patients.

	RTx cases (n = 38)	RTx cases (n = 18)	Controls (n = 16)	p-value
**Immunosuppressive therapy**	**CNI-based**	**CNI-free**		
**Age, years #**	59 ± 12	55 ± 14	57 ± 17	0.692 [1]
**Sex (M/F, %) #**	62/38	67/33	63/38	0.947 [2]
**Current smokers, % #**	19.4	22.2	20.0	1.000 [2]
**Time from RTx, months**	31 (14 – 78)	60 (18 – 96)	n.a.	0.127 [3]
**Enzymatic creatinine, mg/dl**	1.6 (1.4 – 2.0) *	1.7 (1.6 – 2.1) *	0.83 (0.7 – 1.0)	0.000 [1]
**eGFR, ml/min/1.73 m^2**	37.2 (30.3 – 47.6) *	33.7 (32.2 – 46.4) *	89.5 (72.0 – 128.5)	0.000 [1]
**Hypertension, %**	76	72	69	0.827 [2]
**Systolic blood pressure, mmHg**	130 (120 – 140)	125 (120 – 130)	125 (120 – 140)	0.551 [1]
**Diastolic blood pressure, mmHg**	80 (70 – 80)	80 (75 – 80)	80 (70 – 80)	0.760 [1]
**Diabetes mellitus, %**	16	22	25	0.677
**HbA1c, %**	5.7 (5.4 – 6.1) *	5.5 (5.1 – 6.5)	5.2 (4.5 – 5.4)	0.035 [1]
**Hemoglobin, g/dl**	12.9 ± 1.4	12.1 ± 1.4	12.9 ± 1.0	0.087 [1]

**Cholesterol, mg/dl**	221.6 ± 49.0 *	225.5 ± 80.2 *	179.1 ± 37.2 [&]	0.033 [1]
**HDL cholesterol, mg/dl**	64.9 ± 16.4	60.3 ± 13.3	59.9 ± 10.0 [&]	0.651 [1]
**LDL cholesterol, mg/dl**	115.7 ± 47.5	120.8 ± 53.5	97.1 ± 33.4 [&]	0.300 [1]
**Current medication**				
Cyclosporine, n (%)	6 (16)	n.a.	n.a.	n.d.
FK 506, n (%)	19 (50)	n.a.	n.a.	n.d.
MMF, n (%)	n.a.	16 (89)	n.a.	n.d.
Cyclosporine + MMF, n (%)	1 (3)	n.a.	n.a.	n.d.
FK 506 + MMF, n (%)	11 (29)	n.a.	n.a.	n.d.
Sirolimus + MMF, n (%)	n.a.	2 (11)	n.a.	n.d.
Cyclosporine (ng/ml) [&]	91.6 ± 3.2	n.a.	n.a.	n.d.
FK 506 (ng/ml)	8.7 ± 3.1	n.a.	n.a.	n.d.
MMF (ug/ml)	3.6 ± 1.7	4.4 ± 2.7	n.a.	n.d.
ACE inhibitors, %	42.1	61.1	37.5	0.344 [2]
AT1 blockers, %	5.3	11.1	6.3	0.826 [2]
Statins, %	50.0 *	66.7 *	6.3	0.001 [2]

Erythropoietin, %	7.9	22.2	0	0.097 [2]
Steroids, %	94.7	83.3	n.a.	0.314 [2]
**CD34+EPC, units/ml**	75 (25 – 150) *	25 (0 – 100)	24.5 (0 – 25)	0.024 [1]
**CD133+EPC, units/ml**	175 (100 -250) *	200 (75 – 325) *	100 (40-152)	0.021 [1]
**SDF-1, pg/ml**	2679 ± 450 *	2681 ± 437 *	2225 ± 226	0.001 [1]

In cases of normal distribution: variables are presented as mean (±SD). In cases of skewed distribution: variables are presented as median (interquartile range). Comparisons were performed among the three groups. [1]Kruskall-Wallis test; [2]Fisher's exact test; [3]Mann-Whitney test. *p<0.05 compared to control group with Mann-Whitney test or Fisher's exact test. [#]For all data analysis (except for age, sex and current smokers) four patients on calcineurin inhibitor-based therapy are twice measured at different dates and contributed as independent values (34 patients, 38 cases). The term cases characterize all observations included these double measured values. [&]Less than ten non-missing values. ACE, angiotensin-converting enzyme; AT_1, angiotensin-II receptor; eGFR, estimated glomerular filtration rate; HDL, high-density lipoprotein; LDL, low-density lipoprotein; MMF, mycophenolate mofetil; RTx, kidney transplantation; SDF-1, stromal cell-derived factor-1 alpha. (n.a. = not applied; n.d. = not determined).
