# Supplementary material for: Circulating Endothelial Progenitor Cells in Kidney Transplant Patients
Source: PLoS One. 2011 Sep 8;6(9):e24046. doi: 10.1371/journal.pone.0024046 (PMC3169568; doi:10.1371/journal.pone.0024046)
Supplement: Table S2 — Available studies on endothelial progenitor cells (EPCs) after kidney transplantation (RTx). (PDF) [file pone.0024046.s003.pdf]

Table S2. Available studies on endothelial progenitor cells (EPCs) after kidney transplantation (RTx).

Study	EPC identification marker	RTx CNI-based	RTx CNI-free	Anti-hypertensive medication	CKD	Controls	Main Results
**de Groot et al. 2005 [10]**	$CD34^+$	70	4	in 70/74 patients	29	74	In renal transplant recipients EPC numbers were similar to those of healthy controls and significantly higher than in patients with advanced renal failure.
**Soler et al. 2005 [19]**	$CD34^+/CD133^+/CD45^+$	94	0	No information regarding the medication	0	39	Compared to controls the concentration of EPCs is reduced in renal transplant recipients, particularly in those patients with reduced GFR. EPCs show functional impairment after RTx.
**Herbrig et al. 2006 [18]**	$CD34^+$ $CD34^+/CD133^+$ $CD34^+$/VEGF-$R2^+$	20	0	No information regarding the medication	0	22	The amount of $CD34^+$, $CD34^+/CD133^+$, $CD34^+$/VEGF-$R2^+$ cells in the peripheral blood before and after transplantation did not differ. EPC functions improved after transplantation while there number decreased. SDF-1 level decreased after RTx.
**Steiner et al. 2006 [20]**	$CD34^+/KDR^+/CD133^+$	81	24	in 91/105 patients	0	37	EPC did not differ between RTx and healthy individuals. Evidence for a negative association between EPCs and body mass index and blood pressure, whereas statin use was associated with greater EPC counts in stable RTx.
**Metsuyanim et al. 2009 [29]**	VE-C, CD146, CD31, Tie-2, Flk1, CD133 mRNA	8	0	No information regarding the medication	13	10	EPC marker molecules were markedly reduced in ESRD children undergoing hemodialysis compared with control patients. In contrast, transplanted patients exhibited normal gene levels of EPC marker molecules whereas SDF-1 was significantly elevated

Studies identified by Medline and Pubmed searches performed in April 2010. Key words: kidney transplantation and EPC. CKD, chronic kidney disease; CNI, calcineurin inhibitor.
