# Supplementary material for: Circulating Endothelial Progenitor Cells in Kidney Transplant Patients
Source: PLoS One. 2011 Sep 8;6(9):e24046. doi: 10.1371/journal.pone.0024046 (PMC3169568; doi:10.1371/journal.pone.0024046)
Supplement: Table S3 — Clinical characteristics of control and Kidney transplant patients related in Figure S1. (PDF) [file pone.0024046.s004.pdf]

Table S3. Clinical characteristics of control and Kidney transplant patients related in Figure S1.

	**RTx cases (n = 15)**	**RTx cases (n = 14)**	**Controls (n = 11)**
**Immunosuppressive therapy regimen**	**CNI-based**	**CNI-free**	
**Ages, years**	50 ± 12	57 ± 17	41 ± 9
**Sex (M/F)**	11/4	10/4	7/4
**Time from RTx, months**	84 (13 – 192)	27 (10 – 102)	n.a.
**Enzymatic creatinine, mg/dl**	1.8 (1.4 – 2.1)	1.5 (1.1 – 2.0)	1.0 (0.8 – 1.0)
**eGFR, mg/min/1.73 m^2**	38 (34 – 50)	47 (36 – 60)	< 60

In cases of normal distribution: variables are presented as mean (±SD). In cases of skewed distribution: variables are presented as median (interquartile range). CNI, calcineurin inhibitor; eGFR, estimated glomerular filtration rate; RTx, Kidney transplantation. (n.a. = not applied).
